# Exploring Soybean Flower and Pod Variation Patterns During Reproductive Period Based on Fusion Deep Learning

**DOI:** 10.3389/fpls.2022.922030

**Published:** 2022-07-13

**Authors:** Rongsheng Zhu, Xueying Wang, Zhuangzhuang Yan, Yinglin Qiao, Huilin Tian, Zhenbang Hu, Zhanguo Zhang, Yang Li, Hongjie Zhao, Dawei Xin, Qingshan Chen

**Affiliations:** ^1^College of Arts and Sciences, Northeast Agricultural University, Harbin, China; ^2^College of Engineering, Northeast Agricultural University, Harbin, China; ^3^College of Agriculture, Northeast Agricultural University, Harbin, China

**Keywords:** soybean, fusion model, flower, pod, deep learning

## Abstract

The soybean flower and the pod drop are important factors in soybean yield, and the use of computer vision techniques to obtain the phenotypes of flowers and pods in bulk, as well as in a quick and accurate manner, is a key aspect of the study of the soybean flower and pod drop rate (PDR). This paper compared a variety of deep learning algorithms for identifying and counting soybean flowers and pods, and found that the Faster R-CNN model had the best performance. Furthermore, the Faster R-CNN model was further improved and optimized based on the characteristics of soybean flowers and pods. The accuracy of the final model for identifying flowers and pods was increased to 94.36 and 91%, respectively. Afterward, a fusion model for soybean flower and pod recognition and counting was proposed based on the Faster R-CNN model, where the coefficient of determination*R^2^* between counts of soybean flowers and pods by the fusion model and manual counts reached 0.965 and 0.98, respectively. The above results show that the fusion model is a robust recognition and counting algorithm that can reduce labor intensity and improve efficiency. Its application will greatly facilitate the study of the variable patterns of soybean flowers and pods during the reproductive period. Finally, based on the fusion model, we explored the variable patterns of soybean flowers and pods during the reproductive period, the spatial distribution patterns of soybean flowers and pods, and soybean flower and pod drop patterns.

## Introduction

Soybeans are an important food crop all around the world, and are also a major source of oil and protein (Singh et al., 2010). In recent years, many scholars have worked on yield components, such as the number of grains and pods per plant in order to improve soybean yield (Li et al., 2018, 2019; Du et al., 2019; Song et al., 2020). However, the attempts made so far have not fundamentally improved yields (Zhang et al., 2012), so it is essential to find the main factors affecting soybean yields in order to increase them. Soybean flower and pod drops are a common phenomenon during the growth and development of soybeans. It occurs from the emergence of flowers buds to flowering and all stages of podding. The flower and pod drop rates can even reach 30 to 80%. (Selvaraj et al., 2019). Therefore, reducing the flower and pod drop can significantly increase soybean yield (Wang et al., 2010).

In the study of flower and pod drop patterns, many researchers have explored different aspects of the flower and pod patterns of soybeans over the years. Gao et al. (1958) first investigated the order of flower abscission in soybeans and concluded that the order of flower abscission is identical to the order of flowering, with early flower abscission being higher than late flower abscission. Ma et al. (1960) observed average flower drop, pod drop, and bud drop rates of 56.5, 28.4, and 13.1%, respectively, from 163 soybean plants. Song and Dong (2002); Su et al. (2004) conducted an in-depth study on the flowering order, and concluded that the flowering order of limited pod habit soybeans starts from the middle and gradually opens upward and downward, while the flowering order of both sub-limited and unlimited pod habit soybeans opens sequentially from the bottom upward. Zhang et al. (2010) localized the QTL for the number of flowers and pods per plant trait from a genetic perspective and identified gene regions associated with flowering and pods setting. Zhao et al. (2013) broke away from the conventional approach of conducting static observations of flowering and flower drop statistics, and studied flowering patterns from both spatial and temporal perspectives. They concluded that the number of flowers per day varied significantly among different soybean flowering stages, with different flowering nodes and durations. Meanwhile, the drop rate of flowers at different stages also differed significantly. However, there is a gap in this area of research abroad, and, in the studies mentioned above, the indicators were done manually, which can be time-consuming and labor-intensive, and the surveys are subjective, difficult, and error-prone (Bock et al., 2010; Singh et al., 2021). Because of these drawbacks, it is impractical to carry out flower and pod phenotypic surveys manually for large fields and difficult to implement on large samples, thus preventing more general conclusions from being drawn. On the other hand, the complex structure of the soybean plant and the severe shading during the growing season have made many fine phenotypes unavailable, and even important phenomena, such as flower and pod abscission, have been stalled by the difficulty of phenotypic investigations and the lack of real-time, accurate, and bulk phenotypic support for their patterns.

In recent years, there have been significant advances in computer vision technology, largely thanks to the development of neural network techniques, such as deep learning (Lecun et al., 2015). Singh et al. (2021) proposed machine vision as a method to address duress severity phenotyping, which could improve the speed, accuracy, reliability, and scalability of image-based disease phenotyping. Deep learning is a kind of the machine learning method, which predicts complex and uncertain phenomena by learning a large amount of data. DL has led to the pattern transformation of the image-based plant phenotype. This method is not only used for the digital image-based plant adversity phenotype, but also performs well in a wide range of plant phenotype tasks, such as leaf counting (Ubbens et al., 2018), flowering detection (Xu et al., 2018), and plant identification (Šulc and Matas, 2017) with good results (Singh et al., 2018). Gill et al. also introduced the application of machine learning and deep learning methods in plant stress phenotypes. The DL models used for Phenomics mainly include multilayer perceptron, generative antagonism network, convolutional neural network (CNN), and recurrent neural network. CNN has great advantages in image analysis, and different CNN networks are used for different plants (Gill et al., 2022). CNNs in deep learning demonstrate powerful feature extraction capabilities for images and are widely used in image-based agricultural computer vision tasks. In addition, deep learning also plays an important role in field phenotype counting. Many scholars have used deep learning techniques to identify and count different research targets. Rahnemoonfar and Sheppard (2017) proposed a method for estimating fruit counts based on a deep CNN regression model using synthetic data for the training of the network, achieving 91% accuracy on a real data set, which is robust under adverse conditions. Lu et al. (2017) proposed the TasselNet model for counting maize males in complex field environments to estimate them as an output density map. TasselNet has greatly reduced counting errors, but field counting of maize ears is still an open question. Xiong et al. (2019) improved TasselNet by using context-enhanced context-linked local regression networks for field counts of wheat ears, further enhancing the accuracy of TasselNet for field ears by up to 91.01% on the dataset. Hasan et al. (2018) proposed a wheat-counting algorithm based on the Faster R-CNN’s object detection model, but not tested at higher densities. Ghosal et al. (2019) proposed different object detection algorithms for high accuracy detection of sorghum heads in UAV images, all with an accuracy of around 95% and robustness to changing directions, as well as different lighting conditions. Wu et al. (2019) proposed a rice grain-counting method based on Faster R-CNN object detection, with an accuracy higher than 99%, which has a very high accuracy for calculating the number of rice grains per spike. Mu et al. (2020) applied an object detection model to the identification and counting of unripe and ripe tomato fruits at all periods, with an accuracy of 87.83% and good detection accuracy for highly shaded unripe tomatoes in real cultivation scenarios. David et al. (2020) applied the object detection model for experiments on the detection of different periods and varieties of wheat ears, and the results showed that the object detection model has good accuracy, stability, and robustness. Riera et al. (2020) also proposed a combination of object detection and RGB images that could count pods in the R8 period, and thus make a soybean yield prediction. However, they only counted pods in the R8 period, in contrast to our work, where we counted pods from the beginning pod stage to the full maturity stage (R3 to R8). The above methods based on CNNs and regression models focus more on the number of objects present in the image and do not give the position of the objects in the image, and are often used for automatic counting in high-density, high-volume fields; deep networks based on object detection can not only automatically count objects in images but also locale and track them. This shows that the object detection class of models based on deep learning algorithms is more suitable for the localization and identification of soybean flowers and pods.

In this paper, we optimized the Faster R-CNN model for the characteristics of soybean flowers and pods, respectively, and proposed a fusion model based on the Faster R-CNN two-stage object detection algorithm to identify and count flower and pod simultaneously. The fusion model was then used to explore and analyze three aspects of soybean fertility: patterns of flower and pod variation, patterns of flower and pod spatial distribution, and patterns of flower and pod drop in soybeans.

## Materials and Methods

### Experimental Materials

In this paper, four soybean cultivars, namely, DongNong252 (DN252), HeiNong51 (HN51), ZheNong No. 6, and ChunFengZao, were selected as experimental soybean flower samples in three fields in Harbin, Heilongjiang province, Fuyang, Anhui province, and Hangzhou, Zhejiang province, China. The Harbin experimental field in Heilongjiang province is located at an east longitude of 125°42′–130°10′ and north latitude of 44°04′–46°40′, the Fuyang experimental field in Anhui province is located at an east longitude of 114°52′–116°49′ and north latitude of 32°25′–34°04′, the Hangzhou experimental field in Zhejiang province is located at an east longitude of 118°21′–120°30′ and north latitude of 29°11′–30°33′. Among them, DN252 and HN51 have a sub-limited pod habit, while ChunFengZao and ZheNong No. 6 have a limited pods habit. The basic information on image acquisition is shown in Table 1. A total of 1,895 images (3,024 × 4,032 pixels) were captured. The soybean pod test samples were planted at the Northeast Agricultural University experimental field base, and the two cultivars tested were DN252 and HN51. A total of 2,693 images (3,024 × 4,032 pixels) were acquired. Two soybean cultivars, DongNong252 (DN252) and HeiNong51 (HN51), which are mainly grown in Harbin, Heilongjiang Province, were selected as the test samples to study the variation pattern.

**TABLE 1:** Basic information on image acquisition.

Variety	Image acquisition time	Podding habit	Number of images	Color of flower
DN252	2019	Sub-limited podding habit	568	White
ChunFengZao	2020	Sub-limited podding habit	545	White
ZheNong NO. 6	2020	Limited podding habit	266	Purple
HN51	2019	Limited podding habit	516	Purple

In our data samples, the samples with red spider disease are included. Red spider disease damages soybean leaves. At the early stage of damage, yellow and white spots appear on the front of soybean leaves. After 3–5 days, the spot area expands and the spots are dense, and the leaves begin to appear reddish brown patches. With the aggravation of the damage, the leaves turn rusty brown, curl, and, finally, fall off. When spider disease is serious, it will affect the size of soybean flowers and the number of pods. However, at the beginning of suffering from spider disease, we used 2,000–3,000 times of 1.8% avermectin EC to treat the diseased plants. We sprayed the medicine every 5 days, focusing on the back of the upper tender leaves, tender stems, and flower organs of the plants, and sprayed the medicine evenly. After spraying, the soybean plants have completely recovered to normal, and there is no obvious abnormality in flowers and pods, so it has no impact on our identification and counting.

### Image Acquisition and Processing

**FIGURE 1:**
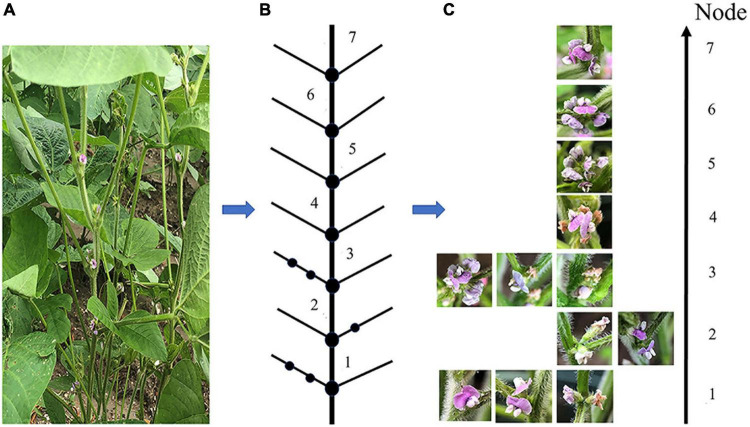
A detailed diagram of the soybean flower data collection scheme. **(A)** Soybean flowers sample plant. **(B)** A structural sketch drawn from the sample plants. **(C)** Images of soybean flowers taken at each node, corresponding to the structural sketch.

Due to soybean plants having a complex structure, various problems made it impossible to obtain a clear picture of all the soybean pods in a single image. These issues include pods and leaves being similar in color, leaves blocking pods and flowers, pods blocking one another, soybean flowers being stacked, flowers too small to see, etc. Nodal shots were used for the soybean flower and pod images. To view the soybean flower characteristics, the viewing angle was overhead, the angle between the phone and the main stem was about 25 degrees, and the distance from the node was 10 to 15 cm; the pods with unobstructed nodes were photographed from a flat angle, and those with heavily obscured nodes from a top angle. The details of the soybean flower data collection method are presented in Figure 1. Figure 1A features a photograph of the flowers at each node on the plant. Figure 1B is a sketch of the sample structure from the overall photograph of the sample plants, with the lowest node number of the soybean structure marked as 1, and the higher nodes numbered sequentially. As seen in Figure 1C, the photographed node soybean flower image corresponds to the individual nodes of the structural sketch.

Flower and pod drop pattern test samples were planted in pots on 25th May 2019 in the Northeast Agricultural University experimental field. Three plants of each variety were selected for observation, and six plants were observed daily at 8 a.m., 12 noon, and 5 p.m. Flowering began on 25th June (R1) and finished on 30th September (R8); the number of flowers per plant, the flowers at the nodes, and the flowers dropped were recorded, and the flowering nodal positions and nodal position of the flowers dropped were photographed. The plants were affected by red spider disease during planting, and, despite being promptly treated with pesticides, some of the sample plants were still affected, rendering it impossible to repeat the trial due to the effects of new crown pneumonia.

The soybean flower and pod datasets were reduced to 640 ×640 pixels using image processing techniques. All datasets were produced in the PASCAL VOC format, and the flowers and the pods in the images were manually labeled as real bounding boxes using the LabelImg tool (Tzutalin, 2018). After production, the datasets were randomly divided into a training set, a validation set, and a test set, with the ratio of the training set plus the validation set to test the set at 8:2. The training and validation sets are then divided in the ratio of 9:1. The specific division of the soybean flower and pod datasets is presented in Table 2.

**TABLE 2:** Classification of the data set for soybean flowers and pods.

Dataset	Division	Number	Dataset	Division	Number
Soybean flower	Training set	1,364	Soybean pod	Training set	1,938
	Validation set	152		Validation set	216
	Test set	379		Test set	539
	Total number	1,895		Total number	2,693

### Models

#### Faster R-CNN (Resnet-50) and Faster R-CNN (VGG16)

Faster R-CNN is a fast, two-stage target detection method, developed after the R-CNN and Fast R-CNN methods, which has been widely used in various fields (Rampersad, 2020). The Faster R-CNN object detection algorithm is divided into four main parts: Feature Extraction Network, Region Proposal Network (RPN), RoI Pooling Layer, and Fully Connected Layer. The network structure is displayed in Supplementary Figure 1A. The RPN replaces the previous Selective Search method and is used to generate candidate boxes, classify and determine whether the set anchor contains the detection target, and perform bounding box regression. RoI Pooling is used to collect the RPN-generated proposals and extract them from the feature maps in the feature extraction network, generating feature maps to be fed into the subsequent fully connected layers for further classification and regression. Finally, we use the proposed feature maps to calculate the specific category and perform another bounding-box regression to obtain the exact final position of the detection box. In this paper, we used both Resnet-50 and VGG16 backbone for the training of the feature extraction network.

#### Single Shot MultiBox Detector

Compared to the two-stage algorithm Faster R-CNN, the first proposed YOLO algorithm has a significantly faster detection speed, but its accuracy rate is inadequate. Consequently, Liu et al. (2016) proposed a Single Shot MultiBox Detector (SSD). The SSD algorithm uses multi-scale features and default anchor boxes to detect the presence of multi-scale objects in the scene in a single step (Akshatha et al., 2022). Its network structure is shown in Supplementary Figure 1B. The SSD network uses a one-stage network to solve the detection speed problem and adopts the anchors idea from Faster R-CNN. Feature extraction is performed in layers and border regression, and classification operations are computed sequentially, allowing training and detection tasks to be adapted to multiple target scales. The elements extracted from the shallow layer help to detect smaller objects, while the deeper layer elements are responsible for detecting larger objects. SSD networks are divided into six stages, each of which learns a feature map and then performs border regression and classification.

#### EfficientDet

EfficientDet, like SSD, is a single-shot detector with an anchor-based target detection method that has achieved good results in both detection speed and accuracy. EfficientNet (Tan and Le, 2019) is the backbone of the network, and the BiFPN feature network extracts the P3, P4, P5, P6, and P7 features in EfficientNet and performs a bidirectional feature fusion. BiFPN (bidirectional feature network) is the core part of the EfficientDet network for fast multi-scale feature fusion (Tan et al., 2020). The fused features are then sent to the class prediction and bounding box prediction networks, which generate the class and bounding box positions of the objects. The class and bounding box network weights are shared between all feature levels. The EfficientDet network structure is presented in Supplementary Figure 1C.

#### YOLOV3

The popular target detection algorithm YOLOV3 (Redmon and Farhadi, 2018) has significant advantages in terms of speed and accuracy (Wang and Liu, 2020). Its backbone network is Darknet53, which uses the 52 layers in front of Darknet-53 (no fully connected layers). This network is a fully convolutional network that makes extensive use of residual hopping connections in order to reduce the negative effect of gradients from pooling, directly removes pooling, and uses the stride in conv to achieve down-sampling. The FPN-like up-sample and fusion approach is used in YOLOV3 to perform detection on multiple feature map scales, improving the detection of mAP and small objects (Redmon and Farhadi, 2018). The network structure is shown in Supplementary Figure1D.

#### YOLOV5

YOLOV5 is the latest algorithm in the YOLO series, and it performs better than other YOLO networks in terms of accuracy and speed (Thuan, 2021). Its network structure, with two different CSPs used in Backbone and Neck, is shown in Supplementary Figure 1E. In Backbone, CSP1_X with a residual structure is used since the Backbone network is deeper and the addition of the residual structure enhances the gradient value when back-propagating between layers. This effectively prevents the gradient from disappearing as the network deepens, resulting in finer features. Using CSP2_X in Neck, the backbone network output is split into two branches as opposed to one simple CBL, which is later concatenated, enhancing the network’s ability to fuse features and retain richer feature information. Furthermore, it uses focus for image slicing operation in Backbone and the FPN+PAN structure in Neck.

#### Fast-SCNN

The Fast-SCNN network consists of 4 parts: a down-sample learning module, a global feature extractor, a feature fusion module, and a classifier (Poudel et al., 2020). The network structure is shown in Supplementary Figure 2A, where it can be seen that the 2 branches share the down-sample learning module to further reduce the computational effort. The learning to down-sample a module consists of 3 convolutional layers, the first of which is a normal convolutional layer and the following two use depth-separable convolution to improve computational efficiency. The global feature extractor is used to extract global features and the learning to down-sample module output feature map is fed into the depth branch of the 2-branched structure. The bottleneck residual block proposed in MobileNet-v2 is used to construct the global feature extractor, where the depth-separable convolution helps to reduce both the number of parameters and the computational effort required (Sandler et al., 2018). The global feature extractor also contains a pyramid pooling module to extract contextual features at different scales (Zhao et al., 2000). The feature fusion module is used to fuse the output features of the 2 branches. Fast-SCNN uses a relatively simple structure for feature fusion to maximize computational efficiency. The classifier module contains two deeply separable convolutions and a convolutional kernel (size, 1 × 1) to improve network performance. SoftMax is also included in the classifier module to calculate the training loss.

#### Image Cascade Network

Image Cascade Network, or ICNet (Zhao et al., 2018), is fed with multiple varying resolution images in order to balance accuracy and speed. Low resolution images are fed into an PSPNet network, called a heavy CNN, where parameters are shared between the branches of low- and medium-resolution images, thus reducing the execution time. High-resolution images are then fed into a light CNN. ICNet throws low-resolution images into a complex CNN and high-resolution images into a lightweight neural network. CFF and cascade label guidance are then used to integrate high resolution features and gradually refine the coarse, low-resolution semantic graph. The ICNet network structure is illustrated in Supplementary Figure 2B.

#### U-Net

The U-Net network structure (Navab et al., 2015) is relatively simple compared to other networks, with Encoder on the left operating as a feature extractor and Decoder on the right operating as an up-sampler. Since the overall network structure is similar to a U shape, it is known as U-Net, and its network structure is shown in Supplementary Figure 2C. The Encoder consists of a convolution operation and a down-sampling operation. The convolution structure is a uniform 3-×-3 convolution kernel with 0 padding and a striding of 1. The above two convolutions are followed by a max pooling with a stride of 2. The output size becomes $\frac{1}{2}\,\left(H,W\right)$. The above steps are repeated 5 times, the final time without max pooling, and the resulting feature map is fed directly into Decoder. The feature map is restored to its original resolution by Decoder, using convolution, up-sampling, and skip connection.

#### DeepLabV3+

The DeepLabV3+ network (Firdaus-Nawi et al., 2011) structure is shown in Supplementary Figure 2D, which adds simple Decoder to DeepLabV3+ to refine the segmentation results, has good target boundary detection results, and is implemented in a two-in-one spatial pyramid pool module or codec structure. In Encoder, DCNN is the backbone network for feature extraction, and Atrous Spatial Pyramid Pooling is based on SPP with Atrous Convolution, which is used for feature extraction with different rates of Atrous Convolution. The features are then compressed through being concat-merged and 1 × 1 convolved. In Decoder, the low-level features are dimensionally adjusted by 1 × 1 convolution and the high-level features are upsampled by a factor of 4 to adjust the output stride. The features are then concat, followed by 3 × 3 convolution, before being upsampled by a factor of 4 to obtain the output prediction.

### Establishment of a Fusion Model

Due to the complex soybean structure, a highly accurate and stable phenotype extraction model is necessary for the process of flower and pod phenotype extraction. Firstly, we selected some classical models to simultaneously identify soybean flowers and pods, and discovered that the Faster R-CNN algorithm object detection was the most accurate, much greater than any other recognition and segmentation models, but there is still room for improvement, so the Faster R-CNN algorithm was fine tuned and a fusion model was finally proposed.

Faster R-CNN is a classical, two-stage, object detection algorithm based on CNNs, which has been widely used in several fields since it was proposed. The model contains four main components: a feature extraction network, a RPN, an RoI pooling layer, and a fully connected layer. The feature extraction network, also known as the backbone network, is generally composed of a CNN that extracts features from the input image to obtain a feature map for use in the proposal module and RoI pooling. Studies have shown that the backbone feature extraction ability largely determines the network performance, and a large deep backbone can effectively improve detection. Therefore, generally speaking, the deeper the network, the better the results, and the more difficult it is to train. However, as the depth increases, so does the network training cost and the effect decreases instead of increasing (He et al., 2016).

The benchmark Faster R-CNN uses a pre-trained VGG16 CNN as the feature extraction network. In the targeting soybean flower recognition and counting section, we used ResNet-50, pre-trained on the ImageNet dataset (Fei-Fei et al., 2010), instead of VGG16 as a feature extraction network to extract deeper flower features as the ResNet-50 residual structure enables the fusion of shallow and deep features, solving the gradient disappearance problem and making the model easier to train.

The backbone selection is particularly critical to soybean pod identification and counting, and four backbone networks were employed: ResNet-50, ResNet-101, CSPResNet-50, and CSPResNet-101, in combination with Feature Pyramid Network (FPN) modules (Lin et al., 2017) for soybean pod identification, whereas Composite Backbone Network was proposed by Liu et al. (2019). The model is composed of different, or multiple, backbones, in a phase-by-phase iterative manner using the output features of the previous backbone as part of the input features for the subsequent backbone, and, finally, using the feature mapping of the last backbone for object detection. The FPN module can fuse lower-level features with less feature semantic information, and higher-level features with more semantic information, to independently perform predictions at different feature levels. Analysis of the results resulted in CSPResNet-50 combined with FPN being used as our pod recognition and counting feature extraction network, where ResNet-50 was chosen as the basic network for the CSPResNet-50 network.

**FIGURE 2:**
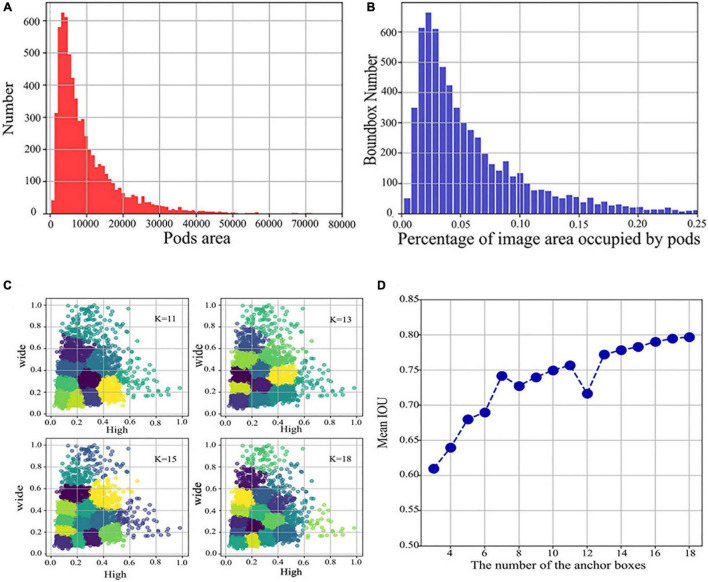
Distribution of pods area and percentage and determining the number of anchor boxes versus the aspect ratio. **(A)** Distribution of the number of different pods areas. **(B)** Distribution of the percentage of the image area occupied by different pods. **(C)** Plots of clustering effects for different *K* values. **(D)** A plot of the number of different anchor boxes versus Mean IOU.

Secondly, according to the characteristics of the large difference in pod size over different periods, we analyzed the pixel area occupied by the 6,334 bounding boxes annotated in the training set, as shown in Figure 2. Figure 2A shows the distribution of the number of bounding box areas, and Figure 2B shows the percentage of the area of the bounding box in the image.

Following the same criteria as the Microsoft COCO dataset (Lin et al., 2014), we took the number of pixels occupied by the bounding box as the area of each pod instance, divided into small, medium, and large scales, according to the following conditions:

1. Small target: the area of pixels occupied by the bounding box ≤32×32;
2. Medium target: 32×32≤ the area of pixels occupied by the bounding box ≤96×96;
3. Large target: the area of pixels occupied by the bounding box ≥96×96.

Taken together, the percentage of annotated bounding boxes in the image ranges from 0 to 0.25, with more significant scale variation, and a large proportion of the annotated bounding boxes are within the small target range. These small targets are likely to be pods at R3 and R4, with multiple scales caused by pod growth variations and different imaging angles. To resolve this issue, we added an FPN module, which extracts multi-scale features from images, enhances semantic information, and strengthens the detection of small target, multi-scale objects.

Suitable anchor boxes can effectively improve accuracy, for example, Mosley et al. (2020) improved detection accuracy by 7% by adjusting anchor boxes based on annotation data, while Zhang et al. (2020) also achieved significant results by modifying anchor boxes in a tomato-disease-detection algorithm based on object detection. This indicates that adjusting the anchor box size can effectively improve the model detection effectiveness. Consequently, we used *k*-means to cluster the normalized labeled bounding boxes in the training set by adjusting the number and size of anchor boxes to make them better fit the pod shape. *k*-means is a common clustering algorithm that allows the input samples to be clustered by similar characteristics into one class. The distance of all points to all the centroids is calculated, and the closest centroid is assigned to the cluster it represents. After one iteration, the centroids are recalculated for each cluster class, and the closest centroid for each point is found again. The procedure is repeated until there is no change in the cluster class for two iterations before and after. Generally, the distance formula selects the Euclidean distance, whereas, here, we take formula (1) to calculate the distance as follows.


(1) d⁢(box,centroid)=1-IOU⁢(box,centroid)

In Eq. 1, intersection over union (IOU), a concept often used in object detection, is used, where it refers to the ratio of the intersection and merge between the box and the centroid. A complete overlap, i.e., a ratio of 1, is ideal, where box denotes the sample box and centroid is the chosen centroid. The larger the overlap area between the sample box and the chosen centroid, the larger its IOU, and so the smaller the 1−IOU, the smaller the distance of the sample from the centroid. To perform clustering, the distance was calculated according to this formula.

Figure 2C shows the clustering plots under different *K* values derived by *k*-means, where the *K* value refers to the number of anchor boxes, and line plots of the relationship between the number of different anchor boxes and Mean IOU are obtained, as in Figure 2D. By investigating the anchor boxes, we found that the Mean IOU changes slowly at 14–18 and almost stops increasing. Therefore, the chosen number of anchor boxes was 18, and all the obtained similar-sized aspect ratios were summed to calculate the mean, resulting in three sets of aspect ratios for testing of 0.75, 1.8, and 3.2.

**FIGURE 3:**
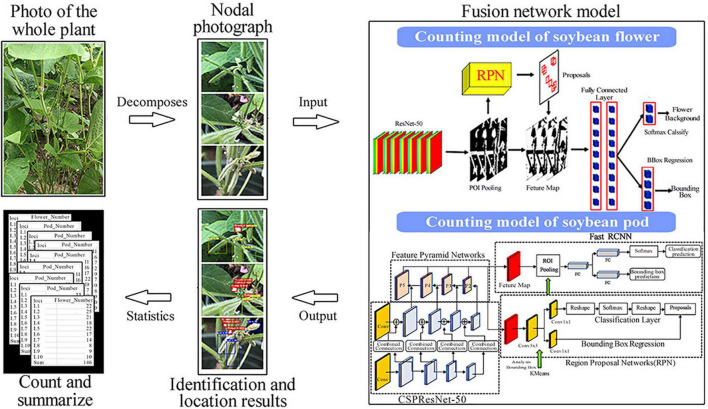
An overall flowchart of the fusion model.

With the above adjustments, we can obtain our fusion model. This is realized by connecting the flower recognition and counting model, and the pod recognition and counting model in series. In the process of image data acquisition, a single plant is taken as the object, so all nodes of the same plant are identified. After identification, a data summary is made. The input image first goes through the flower recognition and counting model to detect the number of flowers, and then into the pod recognition and counting model to detect the number of pods before finally outputting the number of flowers and pods at each node and generating a CSV file, which is summed to the number of flowers and pods per plant. The entire process is shown in Figure 3.

### Flower Drop Rate, Pod Drop Rate, and Pod Formation Rate

The formulae for calculating the flower drop rate (FDR), PDR, and pod formation rate (PFR) are the key to the study of soybean flower and pod drop patterns, and are usually defined as follows, according to the relevant literature (Wang et al., 2014; Xu, 2015).


(2) $$\text{FDR}\left(\%\right)=\frac{\text{TNOFD}}{\text{TNOFD}+\text{TNOPD}+\text{TNOPF}}\times 100\%$$

(3) $$\text{PDR}\left(\%\right)=\frac{\text{TNOPD}}{\text{TNOFD}+\text{TNOPD}+\text{TNOPF}}\times 100\%$$

(4) $$\text{PFR}\left(\%\right)=\frac{\text{TNOPF}}{\text{TNOFD}+\text{TNOPD}+\text{TNOPF}}\times 100\%$$

where FDR = flower drop rate, PDR = pod drop rate, PFR = pod formation rate, TNOPD = total number of flowers dropped, TNOPD = total number of pods dropped, and TNOPF = total number of pods formed. The next step is to determine the soybean plant flower and pod drop rates and explore the soybean flower and pod drop patterns based on the above formulae.

### Hardware and Software

To train CNN on the soybean flower image dataset, we used a personal desktop computer with an Intel (R) Core (TM) i9-9900k CPU, NVIDIA Titan XP (12G) GPU, and 64G RAM under the Pytorch and MXNET deep learning frameworks for the Windows operating systems using Python language to train seven networks. While the soybean pod dataset training was based on PaddlePaddle framework programming, the experiments were trained on an NVIDIA GeForce RTX 2080 Ti GPU with 11 Gb RAM, operating system Ubuntu 14.08, CUDA version v10.1, and Cudnn version 7.6.5.

### Evaluation Indicators

We used the precision, recall, AP, and mAP values to evaluate the results and performance of the different networks used on the dataset, where the precision, recall, AP, and mAP are determined by the following equations:


(5) $$\text{Precision}=\frac{\text{TP}}{\text{TP}+\text{FP}}$$

(6) $$\text{Recall}=\frac{\text{TP}}{\text{TP}+\text{FN}}$$

(7) $$\text{AP}=\sum_{k=1}^{N}\text{Precision}\,\left(k\right)\,△\,\text{Recall}\,\left(k\right)$$

(8) $$\text{mAP}=\frac{\sum_{m=1}^{M}\text{AP}\,\left(m\right)}{M}$$

where TP (True Positive) indicates that the model correctly identified objects from the defined object region, FP (False Positive) indicates that the background was wrongly identified as an object, and FN (False Negative) indicates that the object was wrongly identified as the background. *N* is the total image number in the test dataset, *M* is the total number of categories, *m* is the number of categories, *Precision*(*k*) is the precision value of the *k*th image, and △Recall(*k*) is the change value of the k th image and the *k*−1th image.

Mean intersection over union was used to evaluate the semantic segmentation model. MIOU calculates the ratio of the intersection and the union of the two sets of true and predicted values and can be calculated as follows.


(9) $$\text{MIOU}=\frac{1}{K+1}\,\sum_{i=0}^{K}\frac{\text{TP}}{\text{FN}+\text{FP}+\text{TP}}$$

where TP signifies the prediction is correct, the prediction is positive, and the true is positive; FP denotes that the prediction is wrong, the prediction is positive, and the true is negative; FN denotes that the prediction is wrong, the prediction is negative, and the true is positive; *K* is the total number of categories, and *i* is the number of categories.

## Results

### Selection of a Suitable Counting Model

Firstly, we took the semantic segmentation algorithms U-Net, ICNET, Fast-SCNN, DeepLabV3+, and object detection algorithms Faster R-CNN, YOLOV3, SSD, EfficientDet, and YOLOV5 to detect soybean flowers and pods together, attempting to simultaneously count flowers and pods. For the model evaluation metrics, the object detection algorithm chose the commonly used mAP threshold value of 0.5, and the semantic segmentation algorithm chose MIoU as the evaluation metric. The experimental results are presented in Table 3.

**TABLE 3:** Experimental results of different deep learning algorithms.

Deep learning algorithms	Models	Evaluation indicators	Accuracy
Object detection	Faster R-CNN	mAP	82.63%
	YOLOV3	mAP	68.9%
	YOLOV5	mAP	76.55%
	SSD	MIoU	54.01%
	EffientDet	mAP	80.84%
Semantic segmentation	Fast-SCNN	mAP	54.01%
	ICNET	MIoU	60.02%
	DeepLabV3+	MIoU	56.32%
	U-Net	MIoU	64.42%

The results indicate that the object detection algorithm has a higher detection accuracy than the semantic segmentation algorithm. The highest semantic segmentation algorithm accuracy was 64.42%, and the lowest was 54.01%, both below 70%. The object detection algorithm Faster R-CNN reached an average detection accuracy of 81.7% in the flower and pod counting study, with 85.5% accuracy for flower detection and 77.9% accuracy for pod detection. As can be seen, although the Faster R-CNN has a higher accuracy rate than other object detection algorithms, it still does not meet our requirements for accurately counting flowers and pods simultaneously. Subsequently, the Faster R-CNN algorithm was explored to find a suitable new method, and the Faster R-CNN algorithm was fine-tuned to achieve satisfactory simultaneous counting of flowers and pods. The Faster R-CNN algorithm was then improved for flowers and pods, respectively.

### Training and Evaluation of Six Soybean Flowers and Pods Recognition Models

For the soybean flower counting model, we evaluated the performance of the improved Faster R-CNN (ResNet-50) model and five alternatives [Faster R-CNN (VGG16), SSD, EfficientDet, YOLOV3, and YOLOV5]. The learning rate of these six networks starts at 0.0001, with Faster R-CNN (VGG16), SSD, and Faster R-CNN (ResNet-50), making 200 iterations, EfficientDet, YOLOV3, and YOLOV5 use 50 iterations, with the loss values slowly converged to a value near the exact one. We compared the proposed model with five other methods, based on deep learning, under the same experimental configuration, trained with the same training set, and assessed on the test set. The specific experimental evaluation results are presented in Table 4.

**TABLE 4:** Comparison of different object detection algorithms of flowers and pods.

Flower models	Flower training time (h)	Flower detection accuracy (%)	Pod models	Pod training time (h)	Pod detection accuracy (%)
Faster R-CNN (Resnet-50)	7.89	94.36	Faster R-CNN (CSPResNet-50)	7.19	91%
Faster R-CNN (VGG16)	7.97	82.25	Faster R-CNN (VGG16)	6.88	87.71%
SSD	3.02	90.82	SSD	4.03	84.89%
YOLOV3	0.83	63.96	YOLOV3	1.07	50.45%
YOLOV5	0.95	83.40	YOLOV5	1.07	74.25%
EffientDet	0.93	92.83	EffientDet	0.87	75.09%

It can be observed that the highest of the five alternative models is EfficientDet, with an accuracy of 92.83%, but Faster R-CNN (ResNet-50) also outperformed that by 1.53% on the test set. In terms of training time, the Faster R-CNN (ResNet-50) took longer to train, while the YOLOV3 network took the least time to train. Considering the test accuracy and training time, this study favors Faster R-CNN (ResNet-50) due to its higher detection accuracy and sacrifices training time for accuracy improvement.

For the soybean pod counting model, we evaluated the performance of the improved Faster R-CNN (CSPResNet-50) model and five alternatives [Faster R-CNN (VGG16), SSD, EfficientDet, YOLOV3, and YOLOV5]. The learning rate of these six networks starts at 0.0001, with Faster R-CNN (VGG16), SSD, and Faster R-CNN (CSPResNet-50), making 200 iterations, and EfficientDet, YOLOV3, and YOLOV5 using 50 iterations, with the loss values slowly converged to a value near the exact one. We compared the proposed model with five other deep learning-based methods under the same experimental configuration, trained with the same training set, and assessed on the test set. The specific experimental evaluation results are presented in Table 4.

It can be observed that Faster R-CNN (VGG16) is the highest of the five models, with an accuracy of 87.71%, but Faster R-CNN (CSPResNet-50) also outperformed this by 3.29% on the test set. In terms of training time, the Faster R-CNN (CSPResNet-50) took longer to train, while the YOLOV3 and YOLOV5 networks took the least time to train. When considering the test accuracy and training time, this study favors Faster R-CNN (CSPResNet-50), which has a higher detection accuracy and appropriately sacrifices training time for accuracy improvement.

### Adjusting the Number and Aspect Ratio of Anchor Boxes

All other things being equal, we used CSPResNet-50 as the Faster R-CNN skeleton network for our experiments and used the *k*-means algorithm to adjust the anchor box aspect ratio in the RPN module to 0.75, 1.8, and 3.2. The effect of aspect ratios on the model is discussed, and the experimental results are shown in Table 5. The table shows that following adjustment of the aspect ratio, the overall model detection improved by 0.1% and the small target detection capability improved by 0.5%.

**TABLE 5:** Comparison of test results for adjusting the number of anchor boxes and the aspect ratio.

*k*-means	AP_50_	APs	AP_*M*_	AP_*L*_	FPS
No	91%	26.3%	50.9%	66.3%	17
Yes	91.1%	26.8%	52.3%	67.8%	17

### Evaluation of the Soybean Flowers and Pods Counting Results

**FIGURE 4:**
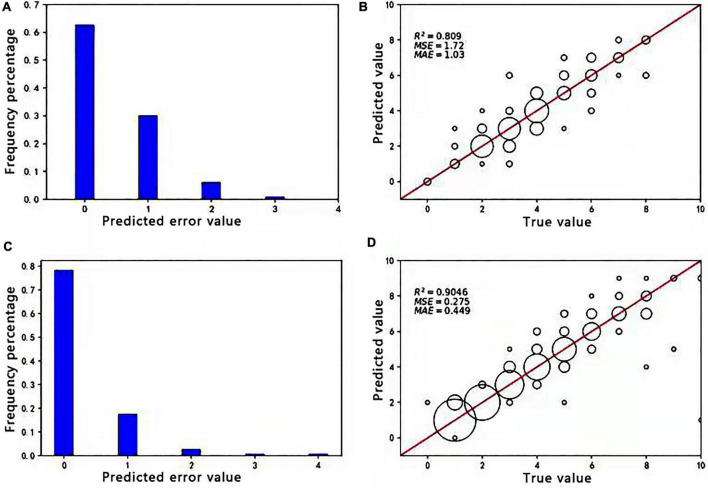
Absolute values of the difference between true and predicted values of soybean flowers and pods and correlation analysis between manual and soybean flower and pod counting. **(A)** Absolute values of the difference between true and predicted values of soybean flowers. **(B)** Correlation analysis between manual and the Faster R-CNN (ResNet50) model for soybean flower counting. **(C)** Absolute values of the difference between true and predicted values of soybean pods. **(D)** Correlation analysis of soybean pod counting by manual and the Faster R-CNN (CSPResNet-50) model.

Approximately, 379 images of soybean flowers were selected along with 539 images of soybean pods, independent of the training sample. The data obtained manually and from Faster R-CNN (ResNet-50) and Faster R-CNN (CSPResNet-50) recognition and counting models were subjected to correlation and error value analyses to evaluate the reliability and accuracy of the models. On this basis, scatter plots and histograms are drawn, as shown in Figure 4. The size of the circle in the diagram is related to the number of times a point appears; the more times it appears, the larger the circle. It can be seen from Figure 4A that 62.7% of the sample set was identified by the flower model with zero errors, 92.7% with no more than one error, and 99.05% with no more than two errors. The percentage of prediction errors of 0 versus 1 for the pod model in Figure 4C is 96%, indicating that the models are still fairly stable. The prediction error value referred to here is the number of differences between the model and manual counts. The coefficient of determination *R*^2^ values for soybean pods, obtained in Figures 4B,D, is 0.809 and 0.9046, respectively, indicating that both Faster R-CNN (ResNet-50) and Faster R-CNN (CSPResNet-50) have good soybean flower and pod counting ability and could replace manual counting.

### Evaluation of the Fusion Model Count Results

**FIGURE 5:**
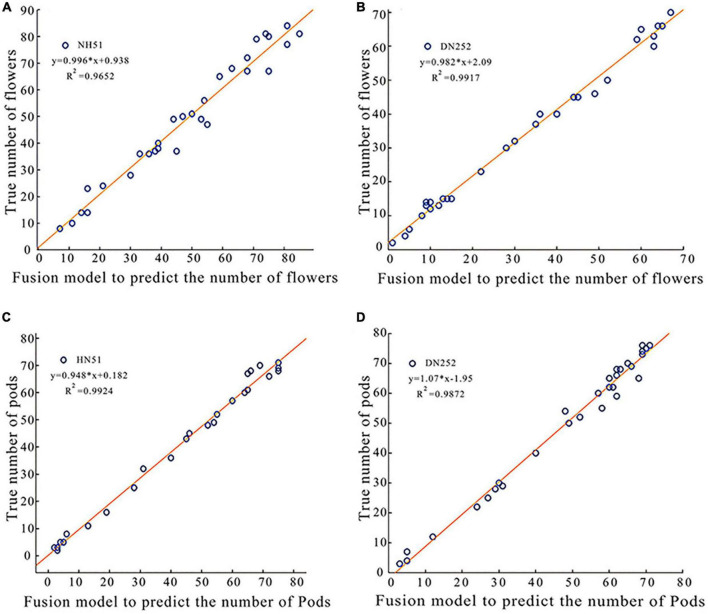
Plots of linear correlation between the fusion model predicted soybean flowers and pods and true values of manual counting. **(A)** A plot of linear correlation between the fusion model-predicted soybean flowers of variety HN51 and true values of manual counting. **(B)** A linear correlation plot of the fusion model predicted the true value of soybean flowers and manual counting for variety DN252. **(C)** A plot of linear correlation between the fusion model-predicted soybean pod of variety HN51 and the true value of manual counting. **(D)** A plot of linear correlation between the fusion model-predicted soybean pod of variety DN252 and the true value of manual counting.

The above exploration reveals that the recognition accuracy of soybean flowers and pods using Faster R-CNN is lower than that of the improved Faster R-CNN (ResNet-50) and Faster R-CNN (CSPResNet-50) separately; thus, a fusion model for soybean flowers and pods is proposed. The detection effect, speed, and accuracy of the fusion model were evaluated. Two soybean plants of two cultivars, DN252 and HN51, were selected as test samples, and the image was fed into a fusion model for flower and pod recognition and counting. The number of flowers and pods per plant was outputted and compared with the manual count, and, on this basis, a linear fit of the fusion model detected pod number versus the actual manual count was plotted, as shown in Figure 5. Figures 5A,B displays the coefficient of determination *R*^2^ values of 0.9652 and 0.9917 for HN51 and DN252 for flowers, respectively. The coefficient of determination*R^2^* values of 0.9924 and 0.9872 for pods is shown in Figures 5C,D. Both are close to 1, indicating that the fusion model performs well at simultaneously counting soybean flowers and pods.

### Study of Flower and Pod Drop Patterns

#### Analysis of Flower and Pod Variation Patterns During the Reproductive Period

**FIGURE 6:**
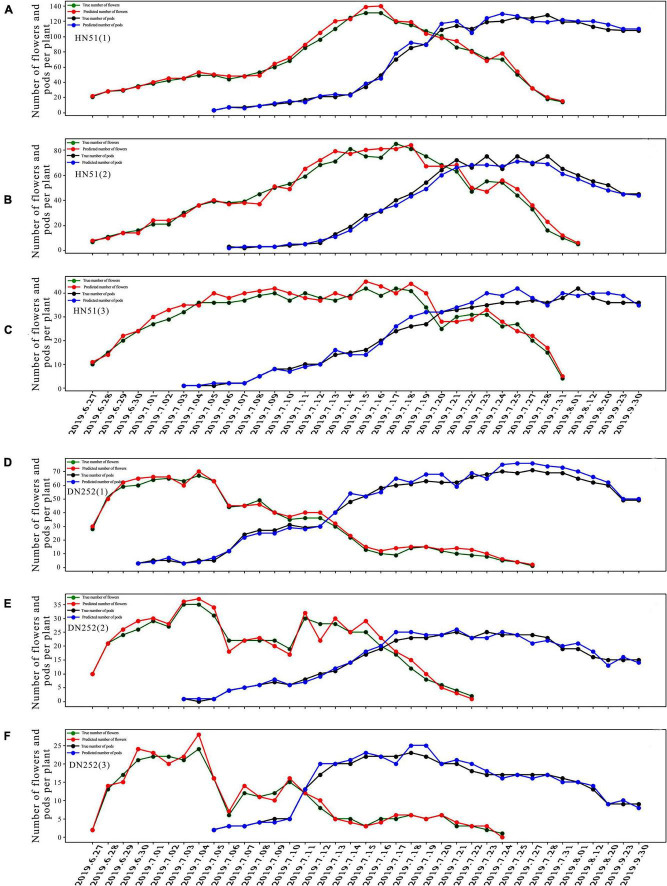
Plots of flower and pod counting over time for three plants of soybean cultivars HN51 and DN252. **(A–C)** Plots of the number of flowers and pods of three plants of variety HN51 over time. **(D–F)** Plots of the number of flowers and pods of three plants of variety DN252 over time.

Using the fusion model, we first analyzed the change patterns of flower and pod drops during the soybean reproductive period, mainly in terms of the number of flowering plants, the number of dropped flowers, and the flowering and pod drop timing. The number of DN252 and HN51 flowers and pods was plotted from 27 June 2019 to 30 September 2019, as shown in Figure 6. HN51 and DN252 have an average daily flowering count of approximately 7.43 and 4.4 and an average daily flower drop of about 3.975 and 7.86, respectively. It can be seen from Figure 6 that different soybean cultivars flower and drop at different rates. HN51 blooms slowly, with a high number of flowers; the peak is shifted backward, and the number of flowers reaches its peak after 20 days of flowering and the duration of the fall is about 10–15 days, as shown in Figures 6A–C. Meanwhile, DN252 blooms quickly, with a maximum of 24 flowers per day on a single plant; the flower number peak is shifted forward, and the number of flowers is low, but the fall is slower and lasts longer, about 20–26 days, as shown in Figures 6D,F. The flowers of both varieties of soybeans last for about a month. The podding timing is similar, with both starting to set around a week after flowering, while HN51 had the fastest podding speed at 3–5 days after peak flowering, gradually reaching a peak, as shown in Figures 6A–C. DN252 starts to set pods at the peak flowering time, and pod numbers reach their peak at mid-fall, as shown in Figures 6D,F. All pods shed after reaching their peak, and slow flowering varieties are more likely to produce high yields in terms of the final number of pods.

#### Analysis of the Spatial Distribution Pattern of Soybean Flower and Pod

**FIGURE 7:**
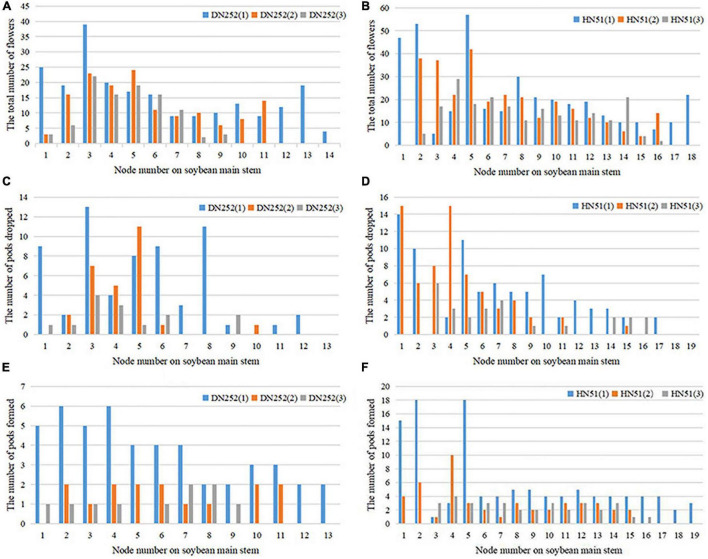
Distribution of total flowers number, number of pods dropped, and number of pods formed at the main stem nodes of the two cultivars DN252 and HN51. **(A)** Distribution of the total number of flowers at the main stem nodes of the DN252 variety. **(B)** Distribution of the total number of flowers at the main stem nodes of variety HN51. **(C)** Distribution of the number of pods dropped at the main stem node of variety DN252. **(D)** Distribution of the number of pods dropped at the main stem node of variety HN51. **(E)** Distribution of the number of pods formed at the main stem node of variety DN252. **(F)** Distribution of the number of pods formed at the main stem nodes of variety HN51.

Next, we investigated the soybean flower and pod spatial distribution pattern. Depending on the podding habit and the number of nodes on the main stem, the distribution can be divided evenly into lower, middle, and upper layers. Figure 7 shows the total number of flowering plants, pods dropped, and pods formed at each main stem node during the reproductive period. The number of flowers on the branch are calculated at the corresponding node. The flower spatial distribution in both soybean cultivars was found to be mainly in the middle and lower layers, with 40.28% of the flowers on the HN51 branch and 37.05% in the middle layer, while the lower DN252 layer accounts for 38.51%, with 40.62% in the middle layer, as shown in Figures 7A,B. The pod drop was observed at all nodes; the HN51 pods drop mainly in the lower and middle zones, while DN252 drops mainly in the middle zone. Both cultivars drop least in the upper zone, as shown in Figures 7C,D. Regarding pod formation, the main DN252 pod-forming areas are the lower and middle layers, while the bulk of HN51 pods forms in the lower layers. This leads to the conclusion that higher pod-forming areas drop more pods and lower pod-forming areas drop fewer pods, as shown in Figures 7E,F.

#### Analysis of Flower and Pod Drop Patterns in Soybeans

The soybean reproductive cycle can be divided into two major growth periods: the first being the V period (Vegetative) and the second being the R period (Reproductive), which can, in turn, be divided into eight reproductive periods: the start of flowering (R1), full bloom (R2), podding starting (R3), full podding (R4), beginning to seed (R5), full seed (R6), beginning to mature (R7), and fully mature (R8; Fehr et al., 1971). The time period covered by this study started at R1 and continued until R8, whereas in previous flower and pod drop studies, rough statistics were presented from the soybean reproductive period, without dividing it into specific reproductive stages. Since it is more convenient to investigate flower and pod drop phenotypes with the proposed fusion model, this is used to conduct statistical analysis during specific reproductive stages. Firstly, the flower drop percentage data between reproductive zones are shown in Table 6, where “period” refers to each soybean reproductive period, “sample” refers to the observed soybean material, and “percentage” denotes the number of flowers dropped by the sample in a given period, as a percentage of the number of flowers dropped during the whole reproductive period. It is found that the FDR varies between cultivars, and the flower drop percentage varies between reproductive stages, varying from time to time but gradually increasing overall. The lowest drop percentage rate is between R1 and R2, with a mean of only 1.9%, while the flower drop peaks between R5 and R6, where it reaches as high as 40%. As the pods grow and develop, the flower drop gradually increases to an average drop rate of 62.49%.

**TABLE 6:** The flower drop rate at different reproductive stages.

Period								
Percentage								
Sample	R1∼R2	R2∼R3	R3∼R4	R4∼R5	R5∼R6	Number of flowers dropped	Total number of flowers	Flower drop rate
DN252(1)	3.62%	12.24%	21.26%	24.88%	38%	110	221	49.77%
DN252(2)	3.50%	15.38%	20.29%	16.78%	44.05%	101	143	70.63%
DN252(3)	2.04%	5.11%	36.73%	26.53%	29.59%	74	98	75.51%
HN51(1)	0.26%	2.06%	45.62%	15.72%	36.34%	196	388	50.52%
HN51(2)	1.02%	5.10%	13.95%	29.59%	50.34%	179	294	60.88%
HN51(3)	0.94%	4.25%	28.80%	26.01%	40.00%	142	210	67.62%
Average value	1.90%	7.36%	27.78%	23.25%	40%	133.67	225.67	62.49%

Table 7 displays the PDR statistics in different reproductive zones, where “period” refers to each soybean reproductive period, “sample” refers to the observed soybean sample, and “percentage” is the number of flowers dropped by a soybean sample in a given period as a percentage of the total number of pods dropped within the entire reproductive period. The data indicate that the PDR varies in different reproductive zones, the pod drop in each reproductive zone, with a trend of increasing and then decreasing with soybean growth. During pod growth, development, and seed filling, the pod drop proportion increases significantly, and the pod drop peaks at the same time as the flower drop peaks, both within R5–R6. Consequently, pod development not only leads to flower drop but also affects the pods themselves. The overall PDR averaged 54.87%.

**TABLE 7:** The pod drop rate at different reproductive stages.

Period								
Percentage								
Sample	R3∼R4	R4∼R5	R5∼R6	R6∼R7	R7∼R8	Number of pods drop	Total number of pods	Pod drop rate
HN51(1)	11.71%	17.11%	23.42%	4.50%	0.00%	111	81	42.18%
HN51(2)	0.00%	11.90%	47.61%	2.38%	2.40%	47	68	59.13%
HN51(3)	8.88%	8.88%	29.17%	12.50%	0.00%	35	33	48.53%
DN252(1)	10.42%	9.90%	18.29%	2.08%	1.56%	48	63	56.75%
DN252(2)	6.96%	13.04%	30.43%	5.22%	3.47%	15	27	64.28%
DN252(3)	7.35%	13.23%	22.06%	1.47%	4.41%	10	14	58.33%
Average value	7.55%	12.34%	28.50%	4.69%	1.97%	44	48	54.87%

## Discussion

### Recognition of Soybean Flowers and Pods in Certain Scenarios

**FIGURE 8:**
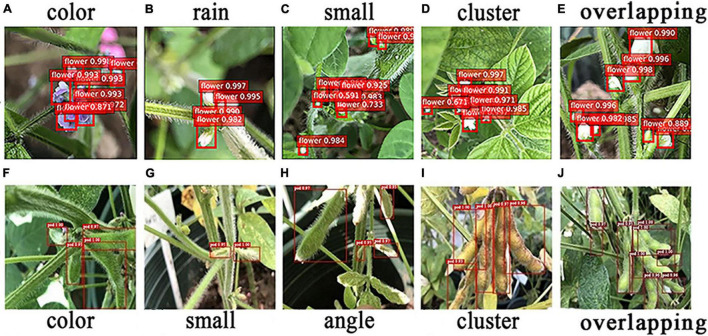
Recognition of flowers and pods in special scenes. **(A–E)** Recognition of flowers in special scenes. **(F–J)** Recognition of pods in special scenes.

Wan and Goudos (2020) utilized Faster R-CNN in the field of fruit image recognition, and the results indicate that it had good, robust detection results in various complex scenarios. Quan et al. (2019) used the Faster R-CNN model to detect maize seedlings in natural environments and demonstrated high performance under various conditions, including full cycle, diverse weather, and multiple angles, which again proved that Faster R-CNN possesses good robustness in complex environments. In this paper, Faster R-CNN (ResNet-50) and Faster R-CNN (CSPResNet-50) models were used to detect flowers and pods in different scenarios, and the detection results are presented in Figure 8.

The five main confounding factors in soybean flower detection are different flower colors, too small flowers, flower clusters, and overlapping flowers. In this study, the soybean flowers were purple and white, and the different colors interfered with the model. Raindrops on the stems and leaves are extremely similar in texture to white flowers when exposed to sunlight, which can cause the model to misinterpret images. In terms of soybean flower size, most are very small targets that are difficult to identify, leading to model miscues. Furthermore, the flowers are mostly clustered together and are indistinguishable, so some may be missed by the model. When the flowers overlap, those at the back are overshadowed by those at the front, and the model may only detect the flowers at the front and miss those at the back. As can be observed in Figure 8, the Faster R-CNN (ResNet-50) model has good flower detection and robustness in all experimental scenarios, as shown in Figures 8A–E.

In addition, as soybean pods are a very similar color to the leaves and stems, the pods may be identified as leaves, etc., leading to errors. The different soybean pod periods can lead to the presence of small targets that the model tends to miss. Differences in camera angles can also lead to incorrect judgments, since the soybean pods may have some shape variation due to the angle of the shot, and some pods will also be obscured and remain unidentified by the model. The soybean pod growth habits, which also bunch together in clusters, can lead to close contact between the pods, and the model may mistake two pods for one, leading to misrecognition. Soybean pods overlap one another, with only a small area being exposed, making it very easy for the model to misidentify them. However, the Faster R-CNN (CSPResNet-50) still had good robustness and good detection results for pods under different environmental situations, such as similar color, small targets, shooting angles, clusters, and overlapping interference, as shown in Figures 8F–J.

### Variation in the Spatial Distribution of Soybean Flowers and Pods Throughout the Reproductive Period

We analyzed the variation in soybean flower and pod spatial distribution throughout the reproductive period, and the results are displayed in Supplementary Figure 3. As seen from section “Analysis of the Spatial Distribution Pattern of Soybean Flower and Pod,” the distribution was divided equally into lower, middle, and upper levels based on the pod setting habit and the number of nodes on the main stem. Finally, we studied the pod drop in all layers and periods, with more pods falling during the period R5–R6 and more dropping in the middle and lower layers compared to the upper layers. The pods formed mainly in the lower layers and dropped more in areas with more pods and less in areas with fewer pods. Soybean flowers are more distributed in the lower and middle layers and fall more where there are more flowers.

We tracked Node 3 and the number of flowers changed from three on 27th June 2019 to four on 3rd July 2019 and then back to three on 12th July 2019. However, one less soybean flower turned into a pod during the period 3rd July 2019 to 12th July 2019. By 17th July 2019, the flowering, flower dropping, and pod formation processes led to three flowers remaining and the pod number increasing to four. One flower was shed by 23rd July 2019, the number of pods remained constant, and one pod was shed between 12th and 20th August 2019. Finally, up until 20th August 2019, the number of pods remained the same before eventually reducing by one.

### Pod Formation Traceability Issues

From the above study, we see that the fusion model has a strong ability to identify and count flowers and pods. Subsequently, we attempted to trace the origin of the pods using the fusion model. This would make it possible to determine which flower a particular pod originates from and to more closely observe the phenotypic changes before and after the flowers are shed. The results are presented in Supplementary Figure 4.

We selected a soybean node in order to detect its temporal image using the fusion model. The sequence indicated by the red arrow is the flower to pod tracking record. The results show that the model does not lose tracking with changes in flower shape or color, nor is it affected by tracking errors due to pod color or growth, different camera angles, or complex backgrounds. This demonstrates it possesses a strong, robust detection capability. However, the failing is that the fusion model only mechanically detects flowers and pods in the image and cannot make causal judgments about them, which still requires human-assisted observation. There is also interference from other flowers and pods in the vicinity, so it is not possible to track and locate individual flowers or pods, which is not suitable for dense environments. We are currently unable to solve this problem, but, in the future, we aim to use a counting method that combines an object detection algorithm with the Deep-Sort algorithm (Wojke et al., 2018) to solve the existing problems in the form of numbered tracking counts of tracked target objects. This would enable flower and pod causal judgments while achieving more detailed origin tracking.

## Conclusion

To investigate soybean flower and pod patterns, we propose a Faster R-CNN-based fusion model for the simultaneous identification and counting of soybean flowers and pods. The main findings are as follows:

1. The improved Faster R-CNN model achieved 94.36 and 91% mAP for soybean flowers and pods, and the results showed that the method can quickly and accurately detect soybean flowers and pods, with strong robustness.
2. The coefficient of determination*R^2^* between the fusion model’s soybean flower and pod counts, and manual counts reached 0.965 and 0.98, indicating that the fusion model is highly accurate in counting soybean flowers and pods.
3. Using the fusion model, we also found the following patterns: firstly, varieties that flower slowly are more likely to produce higher yields; secondly, areas with more pods are more likely to drop pods than areas with fewer pods; thirdly, different varieties have different flower and PDRs and ratios at different stages of fertility, with the flower drop ratio gradually increasing over time and the PDR initially increasing and then decreasing as the soybean grows. The peak range for both flower and pod drops being R5 to R6.

In the future, we will use a combination of object detection algorithms and Deep-Sort algorithms (Wojke et al., 2018) to solve existing counting method issues. We will also locate a platform that allows the automated, high-throughput, and highly accurate acquisition of soybean flower and pod phenotypes to obtain more samples to further demonstrate the patterns we identified.

## Data Availability Statement

The datasets presented in this study can be found in online repositories. The names of the repository/repositories and accession number(s) can be found below: https://pan.baidu.com/s/1j ZE6BHlpVjGay_JqVmOew:password: ate8.

## Author Contributions

RZ: conceptualization, methodology, and software. XW: data curation and writing—original draft preparation. ZY: software, visualization, and data curation. YQ: data curation and software. HT and ZZ: visualization. ZH: visualization and software. YL: supervision and investigation. HZ: investigation and validation. DX: supervision. QC: investigation. All authors contributed to the article and approved the submitted version.

## Conflict of Interest

The authors declare that the research was conducted in the absence of any commercial or financial relationships that could be construed as a potential conflict of interest.

## Publisher’s Note

All claims expressed in this article are solely those of the authors and do not necessarily represent those of their affiliated organizations, or those of the publisher, the editors and the reviewers. Any product that may be evaluated in this article, or claim that may be made by its manufacturer, is not guaranteed or endorsed by the publisher.

## Supplementary Material

Supplementary Figure 1 A network structure diagram of the target detection model. **(A)** A Network structure diagram of Faster R-CNN. **(B)** A network structure diagram of SSD. **(C)** A network structure diagram of EfficientDet. **(D)** A network structure diagram of YOLOV3. **(E)** A network structure diagram of YOLOV5.

Supplementary Figure 2 A network structure diagram of the semantic segmentation model. **(A)** A network structure diagram of Fast SCNN. **(B)** A network structure diagram of U-Net. **(C)** A network structure diagram of ICNet. **(D)** A network structure diagram of DeepLabV3+.

Supplementary Figure 3 Structure of the temporal variation in the number of flowers and pods at different nodes of HN51(3) during the period R1-R8.

Supplementary Figure 4 Tracing the origin of pods.
