# Supplementary figures and images for: Exploring Soybean Flower and Pod Variation Patterns During Reproductive Period Based on Fusion Deep Learning
Source: Front Plant Sci. 2022 Jul 13;13:922030. doi: 10.3389/fpls.2022.922030 (PMC9326440; doi:10.3389/fpls.2022.922030)

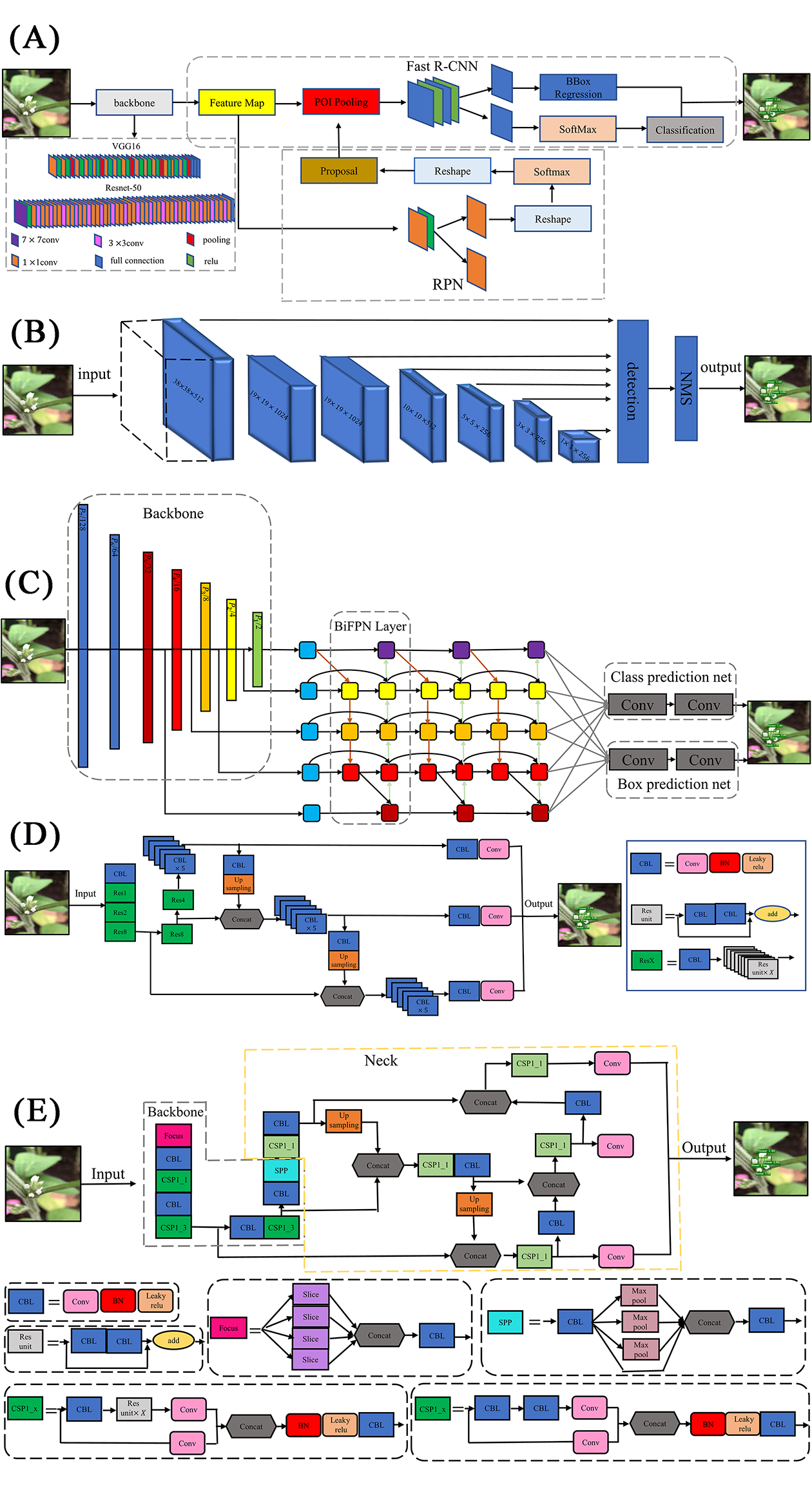

Supplement: Supplementary Figure 1 — A network structure diagram of the target detection model. (A) A Network structure diagram of Faster R-CNN. (B) A network structure diagram of SSD. (C) A network structure diagram of EfficientDet. (D) A network structure diagram of YOLOV3. (E) A network structure diagram of YOLOV5. [file Image_1.TIF]

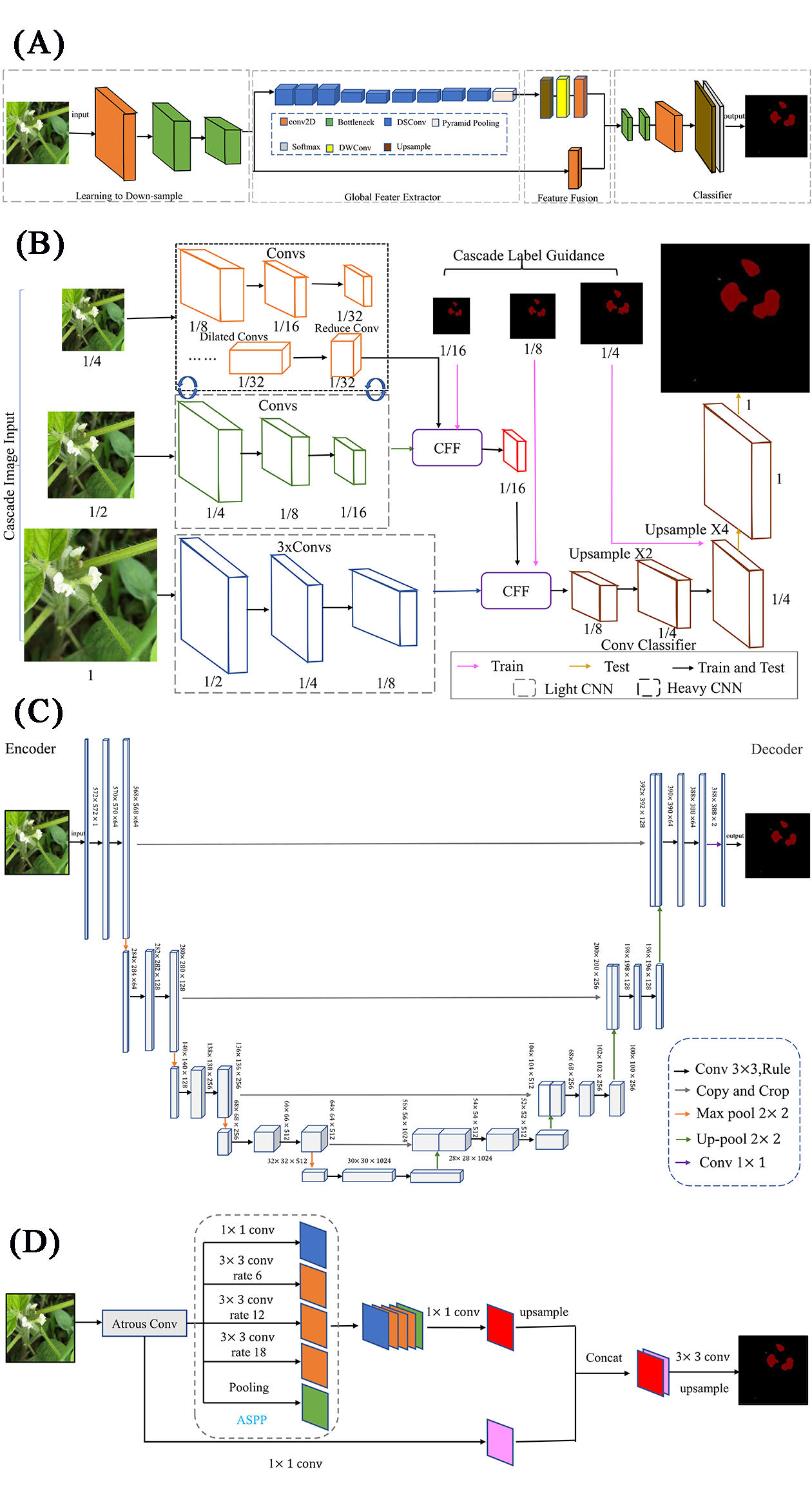

Supplement: Supplementary Figure 2 — A network structure diagram of the semantic segmentation model. (A) A network structure diagram of Fast SCNN. (B) A network structure diagram of U-Net. (C) A network structure diagram of ICNet. (D) A network structure diagram of DeepLabV3+. [file Image_2.TIF]
